# Longitudinal study on morbidity and mortality in white veal calves in Belgium

**DOI:** 10.1186/1746-6148-8-26

**Published:** 2012-03-14

**Authors:** Bart Pardon, Koen De Bleecker, Miel Hostens, Jozefien Callens, Jeroen Dewulf, Piet Deprez

**Affiliations:** 1 Department of Large Animal Internal Medicine, Faculty of Veterinary Medicine, Ghent University, Salisburylaan 133, 9820 Merelbeke, Belgium; 2 Animal Health Service-Flanders, Industrielaan 29, 8820 Torhout, Belgium; 3 Veterinary Epidemiology Unit, Department of Reproduction, Obstetrics and Herd Health, Faculty of Veterinary Medicine, Ghent University, Salisburylaan 133, 9820 Merelbeke, Belgium

**Keywords:** Veal calves, Mortality, Morbidity, Respiratory disease, Bovine viral diarrhea virus, Arthritis, Enteritis, Peritonitis

## Abstract

**Background:**

Mortality and morbidity are hardly documented in the white veal industry, despite high levels of antimicrobial drug use and resistance. The objective of the present study was to determine the causes and epidemiology of morbidity and mortality in dairy, beef and crossbred white veal production. A total of 5853 calves, housed in 15 production cohorts, were followed during one production cycle. Causes of mortality were determined by necropsy. Morbidity was daily recorded by the producers.

**Results:**

The total mortality risk was 5,3% and was significantly higher in beef veal production compared to dairy or crossbreds. The main causes of mortality were pneumonia (1.3% of the calves at risk), ruminal disorders (0.7%), idiopathic peritonitis (0.5%), enterotoxaemia (0.5%) and enteritis (0.4%). Belgian Blue beef calves were more likely to die from pneumonia, enterotoxaemia and arthritis. Detection of bovine viral diarrhea virus at necropsy was associated with chronic pneumonia and pleuritis. Of the calves, 25.4% was treated individually and the morbidity rate was 1.66 cases per 1000 calf days at risk. The incidence rate of respiratory disease, diarrhea, arthritis and otitis was 0.95, 0.30, 0.11 and 0.07 cases per 1000 calf days at risk respectively. Morbidity peaked in the first three weeks after arrival and gradually declined towards the end of the production cycle.

**Conclusions:**

The present study provided insights into the causes and epidemiology of morbidity and mortality in white veal calves in Belgium, housed in the most frequent housing system in Europe. The necropsy findings, identified risk periods and differences between production systems can guide both veterinarians and producers towards the most profitable and ethical preventive and therapeutic protocols.

## Background

The white veal industry is specialized in rearing calves from different breed and origin on a low-iron milk powder diet. The industry is highly integrated and Europe produces about 6 million veal calves yearly, raised predominantly in France, the Netherlands, Italy and Belgium [1]. The incidence of calf diseases differs between production systems and geographical locations and varies over time [2-9]. Therefore collection of local and temporal data is to be preferred. In contrast to conventional dairy, beef, suckler and feedlot calves, mortality and morbidity is hardly documented in veal calves. Previous studies addressed mortality in veal calves housed in individual stalls (crates) in the United States and Canada [10-12]. This housing system has been completely abandoned in Europe (Council Directives 91/629/EC and 97/2/EC) and in certain states in the United States. The only recent European study addressed a niche production system with an exceptionally high animal welfare standard in Switzerland, a minor producing country [13]. White veal production can be divided into three production systems, based upon the type of calf that is reared, namely dairy, beef or crossbred veal. These systems do not only differ according to the selected breeds, but also have a different nutritional and organizational management. Previous studies only addressed dairy calves, whereas in several countries also the other production systems are present [10,13].

No contemporary study on morbidity and mortality in the most frequent veal housing system of the European mainland, which is group housing on slatted floors in pens of 2 to 8 animals after a 6 weeks period of individual housing, is currently available. Nowadays such a study is of particular interest, since multidrug resistance is abundantly present in the veal industry and of great public concern [14-20]. Additionally a recent study showed that antimicrobial use in the veal industry is highest of all food producing animals [21]. All these observations force the veal industry to evaluate current treatment protocols and search for alternative approaches. Longitudinal mortality and morbidity data provide essential information for the understanding of current practices and their consequences in white veal calves, and can form the basis for novel preventive strategies.

Therefore, the objective of the present field study was to determine the causes and epidemiology of mortality and morbidity in white veal calves, housed within different production systems.

## Methods

### Study design, selection of herds and animals

A prospective longitudinal survey based on a sample of 5% of the Flemish veal herds was conducted to monitor all morbidity and mortality events in one production cycle per herd. The study population consisted of all veal herds in Flanders (Northern Belgium) certified by the Belgian Controlled Veal (BCV) label. The sampling frame was the list of veal herds in Flanders officially registered in the Belgian cattle registration system (SANITEL, Animal Health Service-Flanders). Of the 295 herds in Belgium, 285 herds (97%) are situated in Flanders and 271 herds (95%) complied with the BCV label. Because of the intensive registration, the routine visiting and reporting necessary, farms were conveniently selected. Selection criteria included the willingness to keep detailed registration records on diseases and treatment and allowing the use of farm data. Selection was independent of any disease history and for logistic reasons the farms were gradually initiated in the study over a 2 year period. A production cohort was defined as one all in all out production cycle, which lasted from arrival to slaughter (6-8 months). The study group consisted of 15 production cohorts, in 15 herds. The sample was stratified on production system: dairy (n = 5), crossbred (n = 5) and beef (n = 5) cohorts.

### Data collection

#### Registration of mortality data and definitions

All calves were individually identified by ear tag, according to Belgian law. Calf arrival data were collected from the Belgian cattle registration system (SANITEL--Animal Health Service--Flanders). The reasons for calves not finishing the production cycle were death, culling (= unwanted early slaughter) or transfer to another production system. The latter category included calves which were unable to adapt to the intensive milk diet or the concentrate replacer diet and were removed from the veal stables to be fattened as conventional calves. Unwanted early slaughter was defined as calves being individually slaughtered before the rest of the group, mostly for reasons of trauma or sudden respiratory symptoms. Calf identity, mortality date and preceding symptoms were recorded on registration forms by the producers. A gross postmortem examination of the animals which died during production was performed either on farm by a specialized veterinarian or at the Animal Health Service-Flanders. For these postmortem examinations a standardized protocol was always followed. For data processing only one cause of mortality was registered. If more than one lesion was present, the most severe lesion was used as reason of death. If an obvious reason for the animals death was known from the anamnesis (e.g. shock as a consequence of parenteral iron administration) and the autopsy findings complied with this diagnosis, the animal was classified as such. The group of respiratory diseases included two gross diagnoses: pneumonia and necrobacillosis (laryngeal diphtheria). Pneumonia was only held responsible for the animals death if more than one third of the lung was affected, and other lethal lesions were absent. Also pneumonia cases with concurrent pleuritis or pericarditis but without peritonitis (see definition polyserositis underneath) were classified as pneumonia cases. Calves which suffocated as a consequence of air way blockage by necrotic lesions upon the vocal cords/arytenoids were classified as necrobacillosis. The group of digestive diseases included acute ruminal disorders, enteritis, enterotoxaemia, mesenteric torsion, intussusception, liver disease, abomasal hemorrhage and peritonitis due to perforating abomasal ulceration. Ruminal disorders included all acute ruminal pathology (frothy bloat and acute ruminal acidosis/rumenitis) causing sudden death. Cases were classified as enteritis when a macroscopic enteritis and obvious smearing of the hind legs were present at necropsy. Enterotoxaemia was defined as the presence of an extensive necrohaemorrhagic enteritis. Calves were classified as idiopathic peritonitis if at least a peritonitis, without an obvious internal cause (e.g. perforating abomasal ulceration, intussusception,...) was present. This group includes cases in which besides a peritonitis also pericarditis and pleuritis were present (polyserositis). Liver disease included hepatitis, severe hepatic steatosis and generalized icterus. Cases in which the liver was involved in an omphaloflebitis were classified as omphalitis cases, together with omphalitis as such, omphalo-urachitis, omphaloarteritis and umbilical abscesses. Septicemia as such was not determined, and cases which presumably died due to septicemia were classified according to the major lesion (omphalitis, enteritis, meningitis or pneumonia). The group of neurological disorders included hydranencephalia, hydrocephalus and meningitis. The group of orthopedic diseases included euthanasia due to severe arthritis, limb or vertebral column fractures and death due to accidental hanging. Only when necropsied at the Animal Health Service hearts were examined and calves with congenital heart defects were classified as such. Calves which died suddenly and did not show obvious findings of any of the above mentioned acute diseases at necropsy, besides a mild (hemorrhagic) enteritis, were classified as sudden death of unknown origin. Calves which were dead on arrival or died the first day after arrival, were classified as dead on arrival. Calves that were not autopsied (e.g. due to extensive postmortal decay), were classified as such.

#### Detailed necropsies and additional virological and bacteriological investigations

For calves necropsied at the Animal Health Service, histopathology was performed in case no gross diagnosis was possible. Additionally, samples for bacteriological and virological examination were taken. In cases of neonatal enteritis a commercial antigen ELISA for *Cryptosporidium parvum*, bovine coronavirus, bovine rotavirus and *Escherichia coli *(F5) was performed on intestinal content (Digestive ELISA kit, Bio-X, Jemelle, Belgium). Isolation of *Salmonella spp*. was attempted in cases with suspicious lesions, by aerobically culturing intestinal content on brilliant green agar plates (Lab-M, Bury, UK). Isolation of respiratory bacteria (*Pasteurellaceae *and *Mycoplasma spp*.) from pneumonia lesions was performed according to standard protocols, described elsewhere [22-24]. The presence of bovine viral diarrhea virus (BVDV) was examined by PCR on spleen tissue [25]. For selected cases of acute pneumonia PCR analysis for bovine herpesvirus 1 (BHV-1) [26] and bovine respiratory syncytial virus (bRSV) [27] together with virus isolation for bovine adenovirus 3 (BAV-3) and parainfluenzavirus type 3 (PI-3) was performed at the Veterinary and Agrochemical Research Centre (CODA-CERVA, Ukkel, Belgium) according to in house standard protocols, described elsewhere [24].

#### Registration of morbidity data and definitions

Morbidity was estimated on the bases of individual treatment of the calves. A calf was considered a case of a given disease, when treated individually for that indication on at least one day by the producer or veterinarian. This treatment included both single or multiple, antimicrobial or non-antimicrobial drugs. An initial case was defined as the first treatment of a calf for a given indication. A reoccurent case was defined as a calf receiving a new treatment for the same indication more than 5 and less than 15 days after the last treatment for that indication. A relapse case was defined as a calf receiving a new treatment for the same indication more than 14 days after the last treatment for that indication [28]. Data were collected on the bases of the daily recording of individual treatments by the producers on preprinted registration forms. Treatments performed by the veterinarian were also registered on the same forms. The following diagnostic reasons for individual treatment were optioned: (bovine) respiratory disease (BRD), diarrhea, idiopathic peritonitis, acute ruminal disorder, ruminal drinking, otitis, arthritis, omphalitis, laryngeal necrobacillosis, nervous symptoms and miscellaneous. Herds were visited by the primary investigator between 4 and 8 times during the registration period in order to check compliance with the recording system.

### Data management and statistical analysis

Mortality and morbidity (treatment) data were entered in a relational data base (Access 2007, Microsoft Inc., Washington, DC) and transferred to SAS version 9.1 (SAS Institute Inc., Cary, NC) for descriptive and statistical analysis. Mortality data were consistent for 5853 calves. Treatment records (morbidity) were judged as unreliable on 5 cohorts, because of inconsistencies with calf identification. Therefore morbidity data was limited to 3519 calves from 10 cohorts. Mortality/morbidity risks were calculated as the number of mortalities/diseased calves over the number of calves at risk at the start of the study. Mortality/morbidity rates were calculated as the number of mortalities/diseased calves (initial and relapse) over the number of calf-days-at-risk [28,29]. Reoccurent cases (within 14 days after initial treatment) were considered as a failure of initial treatment and therefore not included in the calculations of morbidity risks and rates. For mortality a calf was considered at risk when present alive in the cohort. For morbidity a calf was considered at risk when present alive in the cohort and not individually treated in the past 14 days for the indication of interest [28]. In this respect days spend on oral group antimicrobial treatments were not taken into account. The long acting effect of certain antimicrobial formulations was taken into account by counting one injection as 2 (tilmicosin, amoxicillin, florfenicol, danofloxacin) or 9 days (tulathromycin) of treatment. The proportion of reoccurent and relapse cases was calculated by dividing the number of reoccurent/relapse cases by the number of initial cases for each cohort. In the same way, the fatality rate was calculated as the number of fatal cases of a given disease over the number of initial cases.

Because calves are reorganized according to drinking speed several times per production cycle, analysis at the compartment and pen level was not possible. The unit of analysis was the individual calf. To analyze relationships between production system (dairy, beef or crossbred) and mortality, separate multivariable Cox proportional hazard models were built, with total mortality, pneumonia, enteritis, ruminal disorders, enterotoxaemia, idiopathic peritonitis, death at arrival, abomasal hemorrhage, perforating abomasal ulceration and arthritis as binary outcome variables. The PROC PHREG statement was used, including the positive stable frailty models in the SAS macro to account for clustering within a herd [30]. The end of the observation period was the date of slaughter, if death had not occurred.

To analyze the effect of the production system on morbidity, separate multivariable models were fit with total morbidity, BRD, enteritis, otitis and arthritis as binary outcome variables. PROC GLIMMIX with binomial distribution and logit link function with Wald's statistics for type 3 contrasts was used with herd as random effect. Associations between the different pathological lesions (pneumonia (acute-chronic-pulmonary abscess), enteritis (catarrhal-hemorrhagic), pleuritis, pericarditis, abomasal ulceration, ruminal bloat and peritonitis) and between the lesions and additional diagnostic test results (BVDV PCR and bacteriology of lung lesions) were determined by logistic regression (PROC GLM). Significance was set at *P *< 0.05.

## Results

### Description of study herds composition and management

Herds entered the study between October 2007 and October 2009. A total of 5853 calves was followed (2744 on dairy cohorts, 1624 on crossbred cohorts and 1485 on beef cohorts). Calves on dairy cohorts were predominantly black and red Holstein Friesian (HF), whereas calves on beef cohorts were exclusively Belgian Blue (BB) double muscled calves. In crossbred cohorts, predominantly HFxBB crossbreds were housed. Mean herd size of the selected herds was 679 (standard deviation (SD) = 334), which was comparable to the sampling frame (Student's *t*-test, *P *= 0.22). The average number of calves in the followed cohorts was 390 (SD = 167). The sample contained calves from the three main integrators in Belgium (n = 4797) and from 3 smaller integrators (n = 1056). The 15 herds were under supervision of 5 different veterinary practices. The production cycle length was 196.4 days on average (SD = 9.2; Range (R)(min.-max.) = 174.9-211.0). Calves belonging to the same production cohort were housed in the same stable, which was divided into different compartments in all but two herds. All calves were individually housed during the first 6 weeks and thereafter group housed in galvanized pens on slatted floors. The diet was different between the three production systems. Dairy calves started on a 50/50 ratio with skimmed milk powder and so called nil product (whey and vegetable proteins), which changed to 100% nil product at 8 weeks post arrival on average. Crossbreds received higher quality skimmed milk powder in the first weeks, but eventually also reached a 100% nil product diet. On the contrary, beef calves never reached 100% nil product, and predominantly received high quality skimmed milk powder. In addition, concentrates and fibers were provided in each production system. Calves were not vaccinated against any pathogen.

### Descriptive epidemiology of mortality

**Figure 1:**
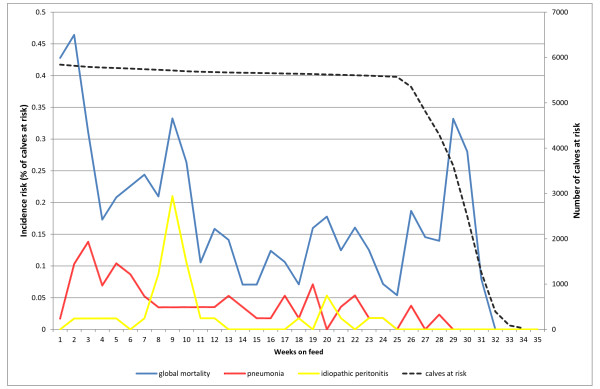
**Mortality risk (%) of pneumonia and idiopathic peritonitis according to week on feed in 5853 white veal calves, housed in 15 cohorts in Belgium (2007-2009)**.

**Figure 2:**
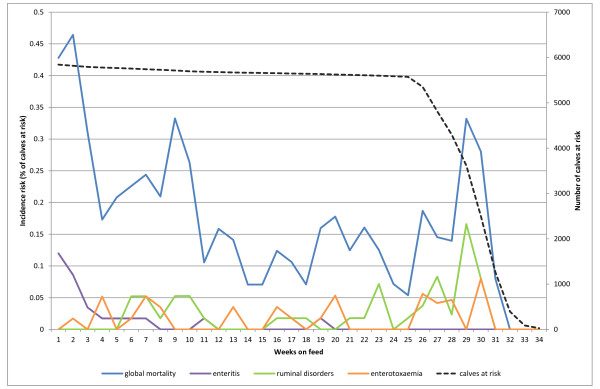
**Mortality risk (%) of selected digestive diseases according to week on feed in 5853 white veal calves, housed in 15 cohorts in Belgium (2007-2009)**.

**Figure 3:**
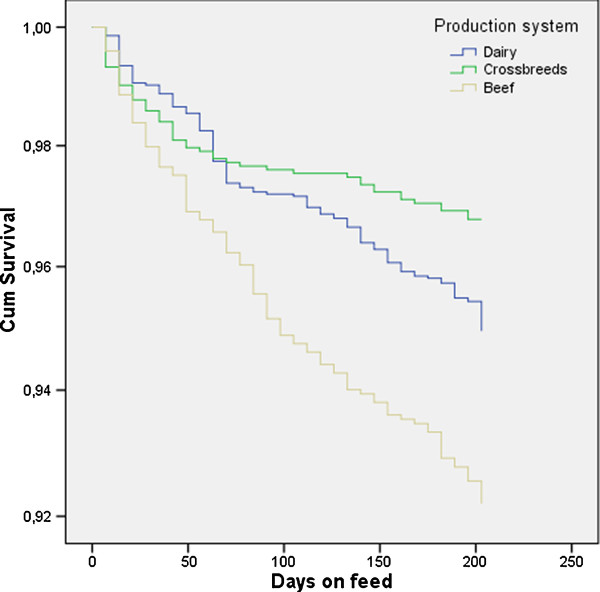
**Survival distribution function for mortality in white veal calves by production system (15 cohorts, 5853 calves, 2007-2009, Belgium)**.

Overall, 308 calves (5.3%) died during production and 0.3% was unwanted early slaughtered. Unwanted early slaughter only occurred on 3 beef cohorts, ranging from 0.6 to 3.8% of the calves. Of the calves that died, on average 82.3% (253/308) was necropsied, ranging from 47.1 to 100.0% at the cohort level. The main reason for not necropsying a calf was the producer neglecting to timely inform the veterinarian, resulting in too advanced postmortal decay for interpretation. The non-necropsied calves also included the calves which were classified as 'death at arrival' (3.6% (11/308)) for the same reason. Overall, the digestive system accounted for 41.9% of mortality, the respiratory system for 27.7%, the musculoskeletal system for 3.6%, the nervous system for 2.0% and idiopathic peritonitis as such for 14.6%. The leading individual causes of mortality were pneumonia (on average 27.0% of the losses at the cohort level; SD = 14.4; R = 7.7-62.5), ruminal disorders (18.6%; SD = 14.4; R = 0-44.4), idiopathic peritonitis (11.9%; SD = 14.3; R = 0-51.5), enterotoxaemia (10.0%; SD = 10.6; R = 0-38.5) and enteritis (9.6%; SD = 8.3; R = 0-25.0%). Mortality risks and rates for the different causes of mortality are listed by production system in Table 1. Overall, the mortality risk was highest in the first weeks after arrival, gradually declined until week 12 and increased again at the end of the production cycle (Figures 1 and 2). Overall, mortality was higher in beef cohorts compared to dairy (hazard ratio (HR) = 1.6; confidence interval (CI) = 1.0-2.5; *P *< 0.05) or crossbred cohorts (HR = 2.3; CI 1.5-3.9; *P *< 0.01) (Figure 3).

**Table 1:** Mortality risk (%) and rates (cases per 1000 days at risk) in white veal calves by production system (15 cohorts, 5853 calves, 2007-2009, Belgium)

Cause of mortality		**Total (n = 5853)**		**Dairy (n = 2744)**		**Crossbreds (n = 1624)**		**Beef (n = 1485)**	
	Percentage of cohorts affected	Mortality risk mean ± SD (min-max)	Mortality rate mean ± SD (min-max)	Mortality risk mean ± SD (min-max)	Mortality rate mean ± SD (min-max)	Mortality risk mean ± SD (min-max)	Mortality rate mean ± SD (min-max)	Mortality risk mean ± SD (min-max)	Mortality rate mean ± SD (min-max)
Total mortality	100.0	5.3 ± 2.5 (1.8-10.9)	0.27 ± 0.13 (0.09-0.58)	4.9 ± 0.8 (3.9-5.7)	0.25 ± 0.030 (0.22-0.28)	3.5 ± 1.6 (1.8-5.3)	0.18 ± 0.08 (0.09-0.27)	7.5 ± 3.0 (4.8 ± 10.9)	0.38 ± 0.16 (0.25-0.58)
Pneumonia	100.0	1.3 ± 1.1 (0.3-3.5)	0.07 ± 0.06 (0.01-0.18)	0.9 ± 0.5 (0.5-1.8)	0.05 ± 0.03 (0.03-0.09)	0.7 ± 0.3 (0.3-1.2)	0.03 ± 0.02 (0.15-0.06)	2.3 ± 1.5 (0.3-3.5)	0.02 ± 0.08 (0.01-0.18)
Ruminal disorders	86.7	0.7 ± 0.6 (0-2.2)	0.04 ± 0.03 (0-0.11)	0.8 ± 0.6 (0.2-1.6)	0.04 ± 0.03 (0.01-0.08)	0.8 ± 0.8 (0.2-2.2)	0.04 ± 0.04 (0.01-0.11)	0.6 ± 0.6 (0-1.2)	0.03 ± 0.03 (0-0.06)
Enterotoxaemia	66.7	0.5 ± 0.7 (0-2.2)	0.03 ± 0.03 (0-0.11)	0.2 ± 0.2 (0-0.4)	0.01 ± 0.01 (0-0.02)	0.2 ± 0.2 (0-0.5)	0.01 ± 0.01 (0-0.03)	1.3 ± 0.7 (0.5-2.2)	0.06 ± 0.04 (0.03-0.11)
Idiopathic peritonitis	73.3	0.5 ± 0.6 (0-2.1)	0.02 ± 0.03 (0-0.10)	0.8 ± 0.8 (0-2.1)	0.04 ± 0.04 (0-1.10)	0.3 ± 0.3 (0-0.6)	0.01 ± 0.01 (0-0.03)	0.3 ± 0.3 (0-0.8)	0.01 ± 0.02 (0-0.04)
Enteritis	80.0	0.4 ± 0.3 (0-1.3)	0.02 ± 0.02 (0-0.06)	0.3 ± 0.3 (0-0.7)	0.02 ± 0.02 (0-0.04)	0.3 ± 0.3 (0-0.6)	0.01 ± 0.01 (0-0.03)	0.6 ± 0.4 (0.2-1.3)	0.03 ± 0.02 (0.01-0.06)
Death at arrival	20.0	0.3 ± 0.7 (0-2.2)	0.01 ± 0.03 (0-0.11)	< 0.1 ± 0.1 (0-0.1)	< 0.01 ± 0.00 (0-0.01)	0.4 ± 1.0 (0-2.2)	0.02 ± 0.05 (0-0.11)	0.3 ± 0.7 (0-1.6)	0.02 ± 0.04 (0-0.08)
Arthritis	26.7	0.1 ± 0.3 (0-1.0)	0.01 ± 0.01 (0-0.05)	0.1 ± 0.1 (0-0.2)	< 0.01 ± 0.01 (0-0.01)	-	-	0.3 ± 0.4 (0-1.0)	0.01 ± 0.02 (0-0.05)
Abomasal hemorrhage	26.7	0.1 ± 0.1 (0-0.3)	< 0.01 ± 0.01 (0-0.02)	0.0 ± 0.1 (0-0.1)	< 0.01 ± 0.00 (0-0.01)	0.1 ± 0.1 (0-0.3)	< 0.01 ± 0.01 (0-0.02)	0.1 ± 0.1 (0-0.3)	0.01 ± 0.01 (0-0.01)
Congenital heart defect	20.0	0.1 ± 0.2 (0-0.5)	< 0.01 ± 0.01 (0-0.03)	0.1 ± 0.2 (0-0.4)	< 0.01 ± 0.01 (0-0.02)	0.2 ± 0.2 (0-0.5)	0.01 ± 0.01 (0-0.03)	-	-
Hydranencephalia	20.0	0.1 ± 0.2 (0-0.6)	< 0.01 ± 0.01 (0-0.03)	< 0.1 ± 0.1 (0-0.1)	< 0.01 ± 0.01 (0-0.01)	0.1 ± 0.2 (0-0.5)	0.01 ± 0.01 (0-0.03)	0.1 ± 0.3 (0-0.6)	0.01 ± 0.01 (0-0.03)
Intussusception	20.0	0.1 ± 0.2 (0-0.5)	< 0.01 ± 0.01 (0-0.028)	0.0 ± 0.1 (0-0.2)	< 0.01 ± 0.01 (0-0.01)	0.1 ± 0.1 (0-0.3)	< 0.01 ± 0.01 (0-0.02)	0.1 ± 0.2 (0-0.5)	0.01 ± 0.00 (0-0.03)
Omphalitis	46.7	0.1 ± 0.2 (0-0.6)	0.01 ± 0.01 (0-0.03)	0.2 ± 0.1 (0.2--0.4)	< 0.01 ± 0.00 (0.01--0.02)	0.1 ± 0.1 (0-0.3)	< 0.01 ± 0.01 (0-0.02)	0.1 ± 0.3 (0-0.6)	0.01 ± 0.01 (0-0.03)
Perforating abomasal ulceration	26.7	0.1 ± 0.2 (0-0.6)	0.01 ± 0.01 (0-0.03)	0.3 ± 0.3 (0-0.6)	0.01 ± 0.02 (0-0.03)	-	-	0.0 ± 0.1 (0.0-0.2)	< 0.01 ± 0.01 (0-0.01)
Unknown sudden death	6.7	0.1 ± 0.5 (0-1.8)	0.01 ± 0.03 (0-0.10)	-	-	-	-	0.4 ± 0.8 (0-1.8)	0.02 ± 0.04 (0-0.10)
Iron shock	13.3	< 0.1 ± 0.1 (0-0.2)	< 0.01 ± 0.00 (0-0.01)	< 0.1 ± 0.1 (0-0.2)	< 0.01 ± 0.00 (0-0.01)	< 0.1 ± 0.1 (0-0.2)	< 0.01 ± 0.01 (0-0.01)	-	-
Liver disease	6.7	< 0.1 ± 0.1 (0-0.5)	< 0.01 ± 0.01 (0-0.024)	< 0.1 ± 0.1 (0-0.2)	< 0.01 ± 0.00 (0-0.01)	-	-	0.1 ± 0.2 (0-0.5)	0.01 ± 0.01 (0-0.02)
Meningitis	6.7	< 0.1 ± 0.1 (0-0.3)	< 0.01 ± 0.00 (0-0.02)	-	-	0.1 ± 0.1 (0-0.3)	< 0.01 ± 0.01 (0-0.02)	-	-
Mesenteric torsion	6.7	< 0.1 ± 0.1 (0-0.3)	< 0.01 ± 0.00 (0-0.016)	-	-	-	-	0.1 ± 0.1 (0-0.3)	< 0.01 ± 0.01 (0-0.02)
Necrobacillosis	13.3	< 0.1 ± 0.1 (0-0.3)	< 0.01 ± 0.01 (0-0.02)	0.0 ± 0.1 (0-0.2)	< 0.01 ± 0.01 (0-0.01)	-	-	0.1 ± 0.1 (0-0.3)	< 0.01 ± 0.01 (0-0.02)
Orchitis	6.7	< 0.1 ± 0.1 (0-0.2)	< 0.01 ± 0.00 (0-0.01)	-	-	< 0.1 ± 0.1 (0-0.2)	< 0.01 ± 0.01 (0-0.01)	-	-
Trauma	13.3	< 0.1 ± 0.1 (0-0.3)	< 0.01 ± 0.01 (0-0.02)	< 0.1 ± 0.1 (0-0.2)	< 0.01 ± 0.01 (0-0.014)	0.1 ± 0.1 (0-0.3)	< 0.01 ± 0.01 (0-0.012)	-	-
Urethra obstruction	6.7	< 0.1 ± 0.1 (0-0.2)	< 0.01 ± 0.01 (0-0.01)	< 0.1 ± 0.1 (0-0.2)	< 0.01 ± 0.01 (0-0.01)	-	-	-	-
Not autopsied	80.0	0.6 ± 0.6 (0-1.8)	< 0.01 ± 0.03 (0-0.09)	0.8 ± 0.6 (0.2-1.6)	0.04 ± 0.03 (0.01-0.08)	0.2 ± 0.3 (0-0.6)	0.01 ± 0.01 (0-0.03)	0.8 ± 0.7 (0-1.8)	0.04 ± 0.03 (0-0.09)

- = no cases

**Figure 4:**
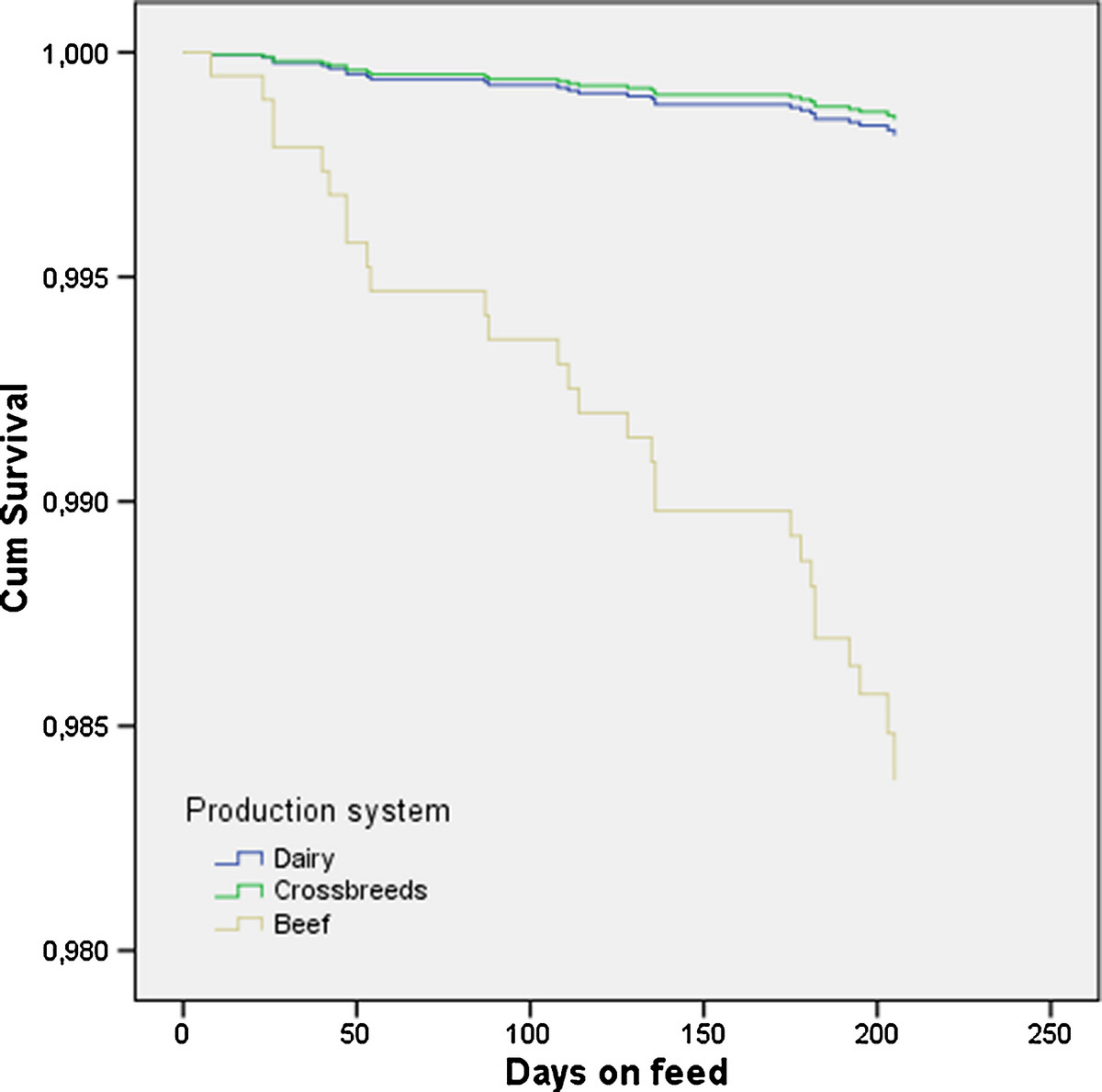
**Survival distribution function for enterotoxaemia in white veal calves by production system (15 cohorts, 5853 calves, 2007-2009, Belgium)**.

Three major peaks in total mortality could be identified (Figures 1 and 2). The first and highest one occurred at week two and in that week the most important causes of mortality were pneumonia (27.3%), enteritis (22.7%), hydranencephalia (13.6%) and omphalitis (13.6%). Mortality due to pneumonia peaked between week 2 and 6, but continued at lower level throughout the entire cycle (Figure 1). Calves housed in beef cohorts were more likely to die from pneumonia compared to dairy calves (HR = 2.5; CI = 1.1-5.8; *P *< 0.05) and crossbreds (HR = 3.2; CI = 1.3-8.0; *P *< 0.05). The second peak, at week 9, was mainly due to idiopathic peritonitis (Figure 1). Idiopathic peritonitis occurred on 66,7% of the studied cohorts. There was no significant influence of the production system on mortality due to idiopathic peritonitis. However, whereas sporadic cases of idiopathic peritonitis occurred in crossbred and beef cohorts, larger outbreaks (0.4 to 2.0% of the calves) occurred in 4 of the 5 dairy cohorts. The third peak was situated at the end of the production cycle and was almost exclusively due to ruminal disorders and enterotoxaemia (Figure 2). Calves housed in the beef production system were much more likely to die from enterotoxaemia than dairy (HR = 7.9; CI = 3.0-20.9; *P *< 0.01) or crossbred calves (HR = 11.6; CI = 2.7-49.7; *P *< 0.01) (Figure 4). Beef calves were also more likely to die from arthritis, compared to dairy calves (HR = 5.6; CI = 1.0-30.5; *P *< 0.05). There was no significant effect of the production system on other causes of mortality. Significant herd effects (*P *< 0.05) were noted for total mortality, pneumonia, ruminal disorders, death at arrival and idiopathic peritonitis.

### Detailed necropsies and additional virological and bacteriological investigations

From 91 necropsied calves (30%; 91/308) additional samples for bacteriology and virology were taken. Of these calves, 57.1% (52/91) showed lesions of pneumonia, 49.5% (45/91) enteritis (21 catarrhal enteritis and 24 hemorrhagic enteritis), 16.5% (15/91) frothy ruminal bloat, 14.3% (13/91) idiopathic peritonitis, 26.4% (23/91) abomasal ulcerations (3 with perforating ulceration and generalized peritonitis), 19.8% (18/91) fibrinous pleuritis, 5.5% (5/91) pericarditis, 4.4% (4/91) congenital heart defects (3 interventricular septum defects, 1 tetralogy of Fallot), 4.4% (4/91) omphalitis (2 umbilical abscesses, 1 omphalophlebitis with liver abscesses and 1 omphaloarteritis) and 3.2% (3/91) an intussusception. Arthritis, meningitis, hydronephros, abomasal displacement, fistulating hepatitis or orchitis each accounted for one calf. Concurrent enteritis and pneumonia occurred in 22 (24.2%) cases, but the association was not significant. Of the 22 young (< 5 weeks old) calves with enteritis, 50%, 18.1%, 13.6% and 4.6% were *Cryptosporidium parvum*, bovine rotavirus, bovine coronavirus and *Escherichia coli F5 *positive respectively. *Salmonella spp*. could not be cultured from any of the examined (n = 13) cases with suspicious lesions.

In 92.3% (48/51) of the pneumonia cases a bacterial bronchopneumonia (of which 9 showed pulmonary abcessation) and only in 7.7% (4/51) an acute interstitial pneumonia was found. Chronic pneumonia was associated with fibrinous pleuritis in 17 (33.3%) cases (odds ratio (OR) = 9.2; CI = 2.9-29.1; *P *< 0.01). Pericarditis was associated with pleuritis (OR = 20.6; CI = 2.1-198.0; *P *< 0.01). From pneumonia cases *Mannheimia haemolytica *(19.4%; 7/36), *Pasteurella multocida *(22.2%; 8/36), *E. coli *(30.6%; 11/36), *Arcanobacterium pyogenes *(25.0%; 9/36) and *Mycoplasma spp*. (33.3%; 11/33) were isolated. Detection of *A. pyogenes *was associated with pulmonary abcessation (OR = 15.6; CI = 2.2-109.8; *P *< 0.01). In 14 cases of acute pneumonia additional virological assays were performed. Of these cases, 35.7% was bRSV PCR positive, whereas BHV-1, PI-3 and BAV-3 were not detected. Overall, 26.0% (19/73) of the examined calves were BVDV PCR positive. A positive BVDV test was associated with chronic pneumonia (OR = 21.6; CI = 5.7-81.9; *P *< 0.01) and pleuritis (OR = 4.9; CI = 1.5-16.3; *P *< 0.01). Of the 13 cases, classified as idiopathic peritonitis, 8 showed concurrent pneumonia, 4 pleuritis and 1 pericarditis. There were no significant associations between the presence of peritonitis on the one hand and pneumonia, pleuritis, pericarditis or abomasal ulcerations on the other hand. Bacteriology of abdominal fluid was performed in 3 cases and yielded two times *M. haemolytica *and once *E. coli*.

### Morbidity

Altogether, 25.4% (896/3519) of the calves developed one or more diseases between arrival and slaughter. The average morbidity risk at the cohort level was 25.0% (SD = 12.9; R = 9.6-45.7) and the morbidity rate was 1.7 calves per 1000 days at risk (SD = 1.0; R = 0.6-3.1). In Table 2 incidence risk and rates of all individually treated diseases are given by production system. BRD occurred most frequently (56.1% of the initial cases), followed by diarrhea (18.5%), scabies (6.3%, exclusively Belgian Blue), otitis (5.7%) and arthritis (5.5%). The proportion of reoccurent cases was on average 9.3% (SD = 8.8; R = 0-24.3) for BRD, 18.8% (SD = 23.9; R = 0-50.0) for necrobacillosis and 0.2% (SD = 0.7; R = 0-2.3) for diarrhea. For other diseases there were no reoccurent cases. The proportion of relapse cases was 10.2% (SD = 6.1; R = 2.4-20.9) for BRD, 31.3% (SD = 47.3; 0-100.0) for necrobacillosis and 1.3% (SD = 4.0; 0-12.5) for arthritis. Case fatality rate was on average 7.8% (SD = 7.5; R = 0-25.0) for BRD, 25.4% (SD = 33.2; R = 0-100) for arthritis, 6.3% (SD = 12.5; R = 0-50.0) for necrobacillosis, 9.4% (SD = 20.6; 0-66.7) for diarrhea and 42.2% (SD = 51.8; R = 0-100.0) for idiopathic peritonitis.

**Table 2:** Incidence risk (%) and rates (cases per 1000 days at risk) of individually treated diseases in 3519 white veal calves by production system (10 cohorts, 2007-2009, Belgium)^a^

Disease		**Total (n = 3519)**		**Dairy (n = 1429)**		**Crossbreds (n = 996)**		**Beef (n = 1094)**	
	Percentage of cohorts affected	Incidence risk mean ± SD (min-max)	Incidence rate mean ± SD (min-max)	Incidence risk mean ± SD (min-max)	Incidence rate mean ± SD (min-max)	Incidence risk mean ± SD (min-max)	Incidence rate mean ± SD (min-max)	Incidence risk mean ± SD (min-max)	Incidence rate mean ± SD (min-max)
Total morbidity^a^	100	31.0 ± 17.4 (11.2-57.3)	1.66 ± 0.97 (0.57-3.14)	26.1 ± 21.8 (11.2-51.1)	1.45 ± 1.23 (0.57-2.86)	26.6 ± 19.3 (15.4-48.8)	1.39 ± 1.05 (0.78-2.60)	38.0 ± 15.2 (20.8-57.3)	2.03 ± 0.88 (1.05-3.14)
Respiratory disease	100	17.9 ± 9.6 (8.2-33.9)	0.95 ± 0.52 (0.41-1.79)	16.8 ± 9.7 (8.3-27.4)	0.93 ± 0.55 (0.42-1.52)	17.3 ± 14.4 (8.2-33.9)	0.90 ± 0.77 (0.41-1.79)	19.1 ± 8.3 (10.8-28.9)	1.00 ± 0.45 (0.58-1.56)
Diarrhea	100	5.7 ± 3.8 (0-0.5)	0.30 ± 0.20 (0.01-0.61)	3.3 ± 3.9 (0.2-7.7)	0.18 ± 0.22 (0.01-0.42)	6.5 ± 2.3 (4.5-9.1)	0.03 ± 0.13 (0.22-0.47)	7.1 ± 4.5 (1.0-11.4)	0.37 ± 0.24 (0.05-0.61)
Arthritis	100	2.0 ± 2.3 (0.2-7.8)	0.11 ± 0.12 (0.01-0.42)	0.8 ± 0.7 (0.2-1.6)	0.04 ± 0.04 (0.01-0.09)	1.0 ± 0.7 (0.3-1.8)	0.05 ± 0.04 (0.02-0.09)	3.7 ± 2.9 (1.1-7.8)	0.19 ± 0.16 (0.06-0.42)
Scabies	20	1.6 ± 4.4 (0-14.1)	0.09-0.24 (0-0.75)	-	-	-	-	4.1 ± 6.7 (0-14.1)	0.22 ± 0.36 (0-0.75)
Otitis	80	1.3 ± 2.2 (0-2.0)	0.07 ± 0.12 (0-0.40)	2.7 ± 4.1 (0-7.4)	0.15 ± 0.22 (0-0.40)	1.0-0.7 (0.3-1.8)	0.05-0.04 (0.02-0.09)	0.4 ± 0.3 (0-0.6)	0.02 ± 0.01 (0-0.03)
Ruminal drinking	40	0.8-1.7 (0-5.4)	0.04 ± 0.09 (0-0.29)	0.7 ± 1.0 (0-1.8)	0.04 ± 0.05 (0-0.10)	-	-	1.5 ± 2.6 (0-5.4)	0.08 ± 0.14 (0-0.29)
Necrobacillosis	40	0.4 ± 0.6 (0-1.6)	0.02 ± 0.03 (0-0.08)	0.1 ± 0.2 (0-0.4)	0.01 ± 0.01 (0-0.02)	-	-	0.8 ± 0.7 (0-1.6)	0.04 ± 0.03 (0-0.08)
Idiopathic peritonitis	30	0.3 ± 0.8 (0-2.6)	0.02 ± 0.04 (0-0.14)	0.9 ± 1.5 (0-2.6)	0.05 ± 0.08 (0-0.14)	0.1 ± 0.2 (0-0.3)	0.01 ± 0.01 (0-0.02)	0.1 ± 0.2 (0-0.3)	< 0.01 ± 0.01 (0-0.02)
Omphalitis	30	0.3 ± 0.7 (0-2.0)	0.02 ± 0.03 (0-0.11)	0.4 ± 0.4 (0-0.7)	0.02 ± 0.02 (0-0.04)	0.7 ± 1.2 (0-2.0)	0.04 ± 0.06 (0-0.11)	-	-
Internal hemorrhage	20	0.2 ± 0.4 (0-1.2)	0.01 ± 0.02 (0-0.07)	0.4 ± 0.7 (0-1.2)	0.02 ± 0.04 (0-0.07)	-	-	0.1 ± 0.1 (0-0.2)	< 0.01 ± 0.01 (0-0.01)
Colic	30	0.1 ± 0.2 (0-0.5)	0.01 ± 0.01 (0-0.03)	-	-	-	-	0.3 ± 0.2 (0-0.5)	0.01 ± 0.01 (0-0.03)
Growth retardation	20	0.1 ± 0.4 (0-1.2)	0.01 ± 0.02 (0-0.06)	-	-	-	-	0.3 ± 0.6 (0-1.2)	0.02 ± 0.03 (0-0.06)
Abcessation	10	0.1 ± 0.2 (0-0.5)	< 0.01 ± 0.01 (0.01-0.42)	-	-	-	-	0.1 ± 0.3 (0-0.5)	0.01 ± 0.01 (0-0.03)
Neurological symptoms	10	0.1 ± 0.2 (0-0.6)	0.01 ± 0.01 (0-0.03)	-	-	-	-	0.2 ± 0.3 (0-0.6)	0.01 ± 0.02 (0-0.03)
Enterotoxaemia	20	0.05 ± 0.1 (0-0.5)	< 0.01 ± 0.01 (0-0.02)	-	-	-	-	0.1 ± 0.2 (0-0.5)	0.01 ± 0.01 (0-0.02)
Trauma	10	0.03 ± 0.1 (0-0.3)	< 0.01 ± 0.01 (0-0.02)	-	-	-	-	0.1 ± 0.2 (0-0.3)	< 0.01 ± 0.01 (0-0.02)
Eye inflammation	10	0.02 ± 0.1 (0-0.2)	< 0.01 ± < 0.01 (0-0.01)	0.1 ± 0.1 (0-0.2)	< 0.01 ± 0.01 (0-0.01)	-	-	-	-
Iron shock	10	0.02 ± 0.1 (0-0.2)	< 0.01 ± < 0.01 (0-0.01)	0.1 ± 0.1 (0-0.2)	< 0.01 ± 0.01 (0-0.01)	-	-	-	-

^a ^Calves could have more than one disease. Both initial and relapse cases were included. - = no cases; SD = standard deviation

**Figure 5:**
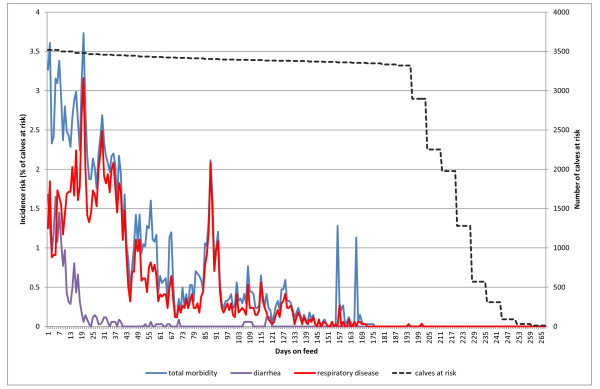
**Incidence risk (%) of individually treated diseases according to days on feed in 3519 white veal calves, housed in 10 cohorts in Belgium (2007-2009)**. A calf was considered morbid on a given day when individually treated with a single or multiple, antimicrobial or non-antimicrobial drug, taking the prolonged effect of the mentioned antimicrobials into account.

Morbidity peaked in the first 3 weeks after arrival, gradually declined and after week 24 hardly any treatments were initiated (Figure 5). There was no significant difference in total morbidity between the production systems. Diarrhea mainly occurred in the first three weeks after arrival. BRD already occurred immediately after arrival, but the peak incidence occurred at week 3 (Figure 5). Only arthritis was significantly more frequently treated in beef cohorts compared to dairy (OR = 5.3; CI = 1.7-16.8; *P *< 0.01) or crossbreds (OR = 3.5; CI = 1.2-10.5; *P *< 0.01). Mange was only a problem in Belgian Blue beef calves. The two peaks near the end of the production cycle (day 156 and 166) were due to individual administration of macrocyclic lactones for mange treatment to a large proportion of Belgian Blue calves in cohort 10. There were no other significant associations between production system and diseases. Significant herd effects were detected for total morbidity and all other assessed diseases.

## Discussion

Random sampling is the method of choice to obtain a representative sample of the population [31]. In the present study herds were conveniently selected based upon motivation, as was done in previous studies to guarantee an adequate follow up and to minimize data loss [3,8,10,13,32-35]. Because the sample size included 5% of the population with more than 90% of the active veterinarians and integrators represented, and because housing and feeding are highly standardized in the Belgian veal industry, the possible selection bias, caused by this selection procedure, is believed to be limited. Because of the convenience selection the sample can only be assumed indicative but not representative for the complete Belgian veal industry at present. The estimation of morbidity was based upon individual treatment by the producer, assuming that treatment rates accurately reflected illness and that increased treatment rates indicated a higher degree of morbidity at that time [10,33]. Antimicrobial use is however influenced by socioeconomic factors and also the personal attitude of the producer might have influenced the difference in treatment rates between the farms [36]. Since easily administrable oral group treatments are frequently used throughout the production cycle, farmers only tended to individually treat calves when severely ill or when only few calves require treatment [21]. In that respect, the individual calf treatments do reflect severe individual calf illness as perceived by the producers.

In the present study, the mortality risk (5,3%) was higher than previously reported for white veal calves in Canada (3,7%), the United States (2,5% and 4,2%) and Switzerland (3,0%) [10,11,13,37]. Including beef cohorts in the present study might explain the higher losses compared to studies on dairy veal calves only, since beef calves are more likely to die [38,39]. However, also the mortality risk within the dairy calves was relatively high (4,9%) compared to previous studies. The most likely explanation is probably the longer production cycle (28 weeks) compared to previously studied systems (16-21 weeks), which increased the days at risk. A second explanatory factor might be the housing system. In the older studies, calves were housed in individual stalls during the complete production cycle. It was postulated that this creates a higher opportunity for individual monitoring and care compared to contemporary group housing [11]. Also the possibility to control feed uptake in individually housed calves might have been a protective factor, since the mortality risk of digestive diseases was far smaller (19,2%) in individual housing, compared to group housing in Belgium and Switzerland (41,9% and 52,0% respectively) [13]. The exact influence of the housing type on mortality remains unclear, since individual housing is nowadays forbidden and previous comparative studies did not report mortality data [40]. Nevertheless, the Swiss study shows that low mortality risks can be achieved in group housing in large pens. However, the fact that in that study, calves were purchased within a day, at a minimum age of three weeks and were housed at low stocking density (> 3,5 m^2^/calf) most likely also contributed to the lower mortality risk.

High mortality risks (8.2%) have been reported in farms which purchase young calves from different origin [41]. Surprisingly, the mortality risk in veal calves was similar to live born calves in dairy replacement herds in Great Britain (5.0%), Norway (4,6%), Sweden (4.0%) and crossbred cow-calf farms in Switzerland (5.0%) and even smaller than reported in large scale dairy calf rearing in Northern America (7,6% and 13,3%) [2,3,7,9,35,42]. Veal producers in Belgium appear to be reasonably able to manage and care for the young, highly stressed calves from multiple origin. However, compared to conventional calf rearing, preventive and metaphylactic antimicrobial drug use plays an important role in this management [21]. Whether the current mortality risk can be maintained with less antimicrobial use is an important question for future research.

Compared to North American (5,5%) or Australian bobby calves (0,6%), transport related mortality was low in Belgian veal calves (0.3%), most likely due to shorter transportation times [43,44]. The finding of hydranencephalia in several dummy calves, was associated with the 2007 bluetongue outbreak in Northern Europe and illustrates how close monitoring of veal calves can assist in the detection of calf diseases of global interest [45]. Diarrhea and related mortality was mainly an issue in the first weeks after arrival, consistent with the risk period in conventional calf rearing [2]. The incidence rate of diarrhea (0,30 cases per 1000 calf days at risk) was smaller than in Swedish (1.17) and North American (1.50) dairy calves, most likely because calves were also monitored in the neonatal period in the latter studies [2,7,8,46]. All major pathogens of the neonatal enteritis complex were found and surprisingly also *E. Coli *F5, suggesting that certain calves were much younger than two weeks old. Although *Salmonella spp*. are historically reported as one of the major causes of mortality in veal calves in Belgium and recent studies still confirmed its presence on Danish veal herds, the bacteria could not be isolated from any of the suspicious cases [47-49]. In contrast to conventional dairy calves, diarrhea and respiratory disease occurred simultaneously in the first three weeks after arrival, which is most likely a consequence of commingling [8,13,50].

BRD was the leading cause of morbidity and mortality. The incidence rate (0,95 cases per 1000 calf days at risk) was similar to Swedish (0.83; measured between birth and 13 weeks of age) or Minnesota dairy calves (1.00; measured between birth and 16 weeks of age), but smaller than in non-weaned Charolais calves in cow-calf herds (1,89; measured between birth and 26 weeks maximum) [2,8,28]. Given the large amount of oral group antimicrobial treatments administered for respiratory disease in veal calves, the incidence is probably severely underestimated and a lot more calves is expected to have suffered from respiratory disease than indicated by individual treatment [51]. Peak incidences of BRD were reached 2 to 6 weeks after arrival, which is at younger age than conventionally housed dairy heifer calves (10 weeks) [2]. Commingling of calves is a major risk factor for BRD, and the peak incidence of respiratory disease is expected immediately after arrival [50,52]. Metaphylactic treatment at arrival, gradual decline of maternal immunity, incomplete maturation of the immune system and the slowly progressive nature of the dominant pathogens in European veal production, namely *Mycoplasma bovis *and BVDV, might have influenced the occurrence of the peak incidence at the age of 1-1.5 months instead of at arrival [24,53-55]. In contrast to cow-calf herds where the BRD incidence remains at a higher level (1.0%), hardly any veal calves still require individual treatment after 3 months of age [2,28]. Most likely the similar age and the all-in all-out management of veal calves limit respiratory disease to the first two months after arrival, whereas in conventional herds pathogens can constantly be transferred from older to younger calves. The long tail of the BRD mortality and morbidity curve is explained by a large proportion of chronic BRD cases (reoccurent and relapse). In addition to previous work, the present study confirms the association of BVDV with chronic pneumonia lesions and pleuritis in white veal calves [24]. As in feedlot calves, the synergy between *M. bovis *and BVDV is the cause of chronic, unresponsive pneumonia, often in association with arthritis and otitis (*M. bovis *associated disease) [24,53,56,57]. In this respect, the higher incidence of arthritis and otitis compared to conventional calves is most likely the consequence of the high prevalence of *M. bovis *in white veal cohorts [8]. In the present study crossbreds had marked lower mortality due to respiratory disease. This heterosis effect has also been observed in other production systems [38,39,58].

As mentioned earlier, digestive diseases were an more important cause of mortality in the recent studies on group housed calves, compared to an older study on individually housed calves [10,13]. In group housed veal calves in Switzerland, much more calves died from perforating abomasal ulceration (0.53% vs. 0.11% in the present study) and intestinal torsion (0.4% vs. 0.02%) compared to group housed calves in Belgium [13]. In contrast very few calves (0.14%) died from ruminal bloat in Switzerland, whereas ruminal bloat (0.7%) and enterotoxaemia (0.5%) were the most important digestive causes of mortality in Belgium [13]. Although both diseases occurred throughout the production cycle, the main risk period was situated near the end of the production round, when feed uptake was at its highest. In contrast to ruminal disorders, enterotoxaemia almost exclusively occurred in Belgian Blue veal calves. The causative agent is *Clostridium perfringens*, but the identity of the toxin and the exact pathogenesis are still unclear [59,60]. Also Belgian Blue suckler calves are highly susceptible for enterotoxaemia, and it is unclear whether there is a breed predisposition or whether dietary differences between the studied production systems are the cause [61].

Finally, one of the most remarkable causes of mortality in the present study, was idiopathic peritonitis, especially in dairy veal calves. Idiopathic peritonitis emerged only recently in veal calves and the peak incidence at week 9, shortly after the respiratory problems, suggests septicemic spread of bacteria from the lungs to the peritoneum. In one outbreak in Belgium *P. multocida *capsular type F has been isolated from peritoneal fluid in two cases [62]. Also, *P. multocida *capsular type B was isolated from outbreaks of pleuritis and peritonitis in intensive dairy calf rearing facilities in New Zealand [63]. In the present study no significant association between pneumonia and peritonitis could be demonstrated at necropsy and only *M. haemolytica *and *E. coli *could be isolated from peritoneal fluid. Given these contradictory necropsy results, and the identification of a specific risk period in the present study, more research is necessary to identify the aetiology of idiopathic peritonitis.

## Conclusions

The present study offers a benchmark for morbidity and mortality data in the most common housing system for white veal calves in Europe, based upon the Belgian situation. Respiratory disease was the leading cause of morbidity and mortality. BVDV was associated with chronic pneumonia and pleuritis at necropsy. Calves housed in beef cohorts were at higher risk to die from pneumonia, enterotoxaemia and arthritis. This information can be used to evaluate preventive and therapeutic protocols and can direct producers towards the most profitable strategy with attention for public health and animal welfare.

## Competing interests

The authors declare that they have no competing interests.

## Authors' contributions

Conception and design of the study: BP, KDB and PD; Farm visits and follow up: BP, KDB; post-mortem examinations: JC; Data acquisition and statistical analysis: BP, JD, MH; Drafting and critically revising the manuscript: BP, JD, PD. All authors read and approved the final manuscript.
